# Holobionts and ecological speciation: the intestinal microbiota of lake whitefish species pairs

**DOI:** 10.1186/s40168-018-0427-2

**Published:** 2018-03-14

**Authors:** Maelle Sevellec, Nicolas Derome, Louis Bernatchez

**Affiliations:** Département de Biologie, Institut de Biologie Intégrative et des Systèmes (IBIS), Université Laval, 1030, Avenue de la Médecine, Québec, Québec G1V 0A6 Canada

**Keywords:** Whitefish intestinal microbiota-speciation

## Abstract

**Background:**

It is well established that symbionts have considerable impact on their host, yet the investigation of the possible role of the holobiont in the host’s speciation process is still in its infancy. In this study, we compared the intestinal microbiota among five sympatric pairs of dwarf (limnetic) and normal (benthic) lake whitefish *Coregonus clupeaformis* representing a continuum in the early stage of ecological speciation. We sequenced the 16s rRNA gene V3-V4 regions of the intestinal microbiota present in a total of 108 wild sympatric dwarf and normal whitefish as well as the water bacterial community from five lakes to (i) test for differences between the whitefish intestinal microbiota and the water bacterial community and (ii) test for parallelism in the intestinal microbiota of dwarf and normal whitefish.

**Results:**

The water bacterial community was distinct from the intestinal microbiota, indicating that intestinal microbiota did not reflect the environment, but rather the intrinsic properties of the host microbiota. Our results revealed a strong influence of the host (dwarf or normal) on the intestinal microbiota with pronounced conservation of the core intestinal microbiota (mean ~ 44% of shared genera). However, no clear evidence for parallelism was observed, whereby non-parallel differences between dwarf and normal whitefish were observed in three of the lakes while similar taxonomic composition was observed for the two other species pairs.

**Conclusions:**

This absence of parallelism across dwarf vs. normal whitefish microbiota highlighted the complexity of the holobiont and suggests that the direction of selection could be different between the host and its microbiota.

**Electronic supplementary material:**

The online version of this article (10.1186/s40168-018-0427-2) contains supplementary material, which is available to authorized users.

## Background

Earth is dominated by approximately 10^30^ microbial cells [1], which is two- or three-fold more than the number of plant and animal cells combined [2]. Therefore, it is important to consider that animal and plant evolution has and continues to occur in the presence of microbiota, which have either parasitic, mutualistic, or commensal interaction with a host [3]. The ubiquity and importance of the microbiota is supported by its influence on host development, immunity, metabolism, behavior, and numerous other processes including speciation [4–12]. The host (animal or plant) and their microbiota are referred to as a “holobiont” [10, 13–15], which represents a unique biological entity evolving through selection, drift, mutation, and migration [16].

The concept of holobiont offers a new angle for the study of adaptive divergence ultimately leading to speciation. For instance, the role of microbiota on pre-zygotic isolation has recently been documented [17]. Moreover, the host’s visual, auditory, and chemosensory signals implicated in mate choice could be influenced by its microbiota [18–22]. Also, host populations sharing similar environment or diet have been shown to share similar microbiomes, known as a “socially shared microbiome” [17]. The socially shared microbiome could recognize specific signals of the host population and thus influence its evolution in ways that are microbe-specific and microbe-assisted, which may lead to post-zygotic isolation [17].

The intestinal microbiota could be particularly prone to playing a key role in the process of population divergence and speciation given its broad array of functional impacts on its host [23]. The involvement of the intestinal microbiota in organismal functions comprises nutrition [24, 25], toxicity resistance [26], energy metabolism [9, 27, 28], morphology [29], and behavior [5, 8, 30, 31]. On the other hand, the intestinal microbiota can also promote host phenotypic plasticity, which may contribute to adaptation. For example, new intestinal microbiota genes can be acquired from the environment through acquisition of new bacteria [32, 33]. The intestinal microbiota can also adapt in response to variation in the host’s physiological and environmental conditions [34]. Moreover, the short generation time of the intestinal microbiota and the horizontal transfer of genes can favor rapid microbiota evolution [35, 36].

While there are now a plethora of studies that have documented the positive influence of holobionts on hosts, including humans, relatively few studies have focused on fish microbiota in the wild even though they represent around 50% of the total vertebrate diversity [37, 38]. To date, about 20 studies have investigated fish intestinal microbiota in the wild (e.g., [39–43]). Of these, very few concerned speciation and to our knowledge, none analyzed specifically the adherent bacteria present in the fish epithelial mucosa [44–49]. Adherent bacteria are of particular interest because they may interact more closely with their host than bacteria present in the alimentary bolus [47].

Lake whitefish (*Coregonus clupeaformis*) comprises sympatric species pairs referred to as dwarf and normal whitefish that are found in five lakes of the St. John River drainage in the province of Québec, Canada, and in Maine, USA. A relatively recent period of post-glacial adaptive radiation occurred approximately 12,000 years before present (YBP), leading to parallel phenotypic and ecological divergence in different lakes of the dwarf whitefish derived from the ancestral normal whitefish [50]. Dwarf and normal whitefish are partially reproductively isolated in each lake [51], differ in genetically based morphological, physiological, behavioral, ecological, and life history traits [52–56] and occupy the limnetic and benthic habitat, respectively. Dwarf and normal whitefish also differ in trophic niche, where dwarf whitefish (and limnetic whitefish in general) feed almost exclusively on zooplankton [57, 58] and normal whitefish are more generalist and feed on more diverse prey items including zoobenthos, molluscs, and fish prey [50, 58].

In this study, we investigate the within- and between-lake variation in the intestinal microbiota among these five sympatric pairs of dwarf and normal whitefish, representing a continuum in the early stage of ecological speciation. We sequenced the 16S rRNA gene of adherent bacteria present in the intestinal tissue and in order to test for differences between intestinal microbiota of dwarf and normal whitefish pairs. We chose adherent microbiota present on intestinal tissues because this microbiota may be more involved in host-microbiota interactions. In parallel, we also sequenced the 16S rRNA gene of water bacterial communities from the five lakes in order to test the association between the water bacterial community and the whitefish intestinal microbiota. Ultimately, our main goal was to test for the occurrence of parallelism in the microbiota of sympatric dwarf and normal whitefish across different environments, where evidence for parallelism would provide strong indirect evidence for the role of natural selection in shaping host microbiota.

## Methods

### Sample collection

Lake whitefish (44 dwarf and 64 normal fish) were sampled with gill nets from Cliff Lake, Indian Pond, and Webster Lake in Maine, USA, in June 2013, and from East and Témiscouata lakes in Québec, Canada, during summer 2013, from May to July (Table 1). Fish were dissected in the field in sterile conditions. The ventral belly surface was rinsed with 70% ethanol, and non-disposable tools were rinsed with ethanol and flamed over a blowtorch between samples. The intestine was cut at the hindgut level (posterior part of the intestine), and the digesta were aseptically removed. Then, the intestine was cut at the foregut level (anterior part of the intestine), removed from the peritoneal cavity, and clamped on both extremities in order to isolate the adherent bacteria in the laboratory. The clamped intestines were individually stored in sterile cryotubes and flash-frozen in liquid nitrogen. Water samples (2 L) were collected in each lake at four depths (at the top of the water column, at 5, 10, and 15 m corresponding to 1 m above the lake bottom) with a Niskin© (General Oceanics). Water samples were filtered first with a 3.0-μm mesh, followed by a 0.22-μm nitrocellulose membrane using a peristaltic pump (Cole-Parmer: Masterflex L/S Modular Drive). The 0.22-μm membranes were placed into cryotubes and flash-frozen with liquid nitrogen. All samples were transported to the laboratory and kept at − 80 °C until further processing.

**Table 1:** Number and location of samples, sampling dates, *F*_ST_, and core microbiota for each species in each lake

Lakes	Species/water	Normal-dwarf pairwise	Number of fish intestinal mucosa	Percent of shared sequences	Water samples at different depths	Sampling date	Localization
		*F*_ST_					
Cliff	D	0.28	12	35.7	–	June 13–14, 2013	46°23′59″N, 69°15′11″W
	N		12	51.6	–		
	T		24	–	–		
	W		–	–	6		
East	D	0.02	8	60.4	–	July 2–4, 2013	47°11′15″N, 69°33′41″W
	N		12	39.2	–		
	T		20	–	–		
	W		–	–	8		
Indian	D	0.06	11	64.9	–	June 10–11, 2013	46°15′32″N, 69°17′29″W
	N		15	36.1	–		
	T		26	–	–		
	W		–	–	8		
Témiscouata	D	0.01	10	44.5	–	May 28–30, 2013	47°40′04″N, 68°49′03″W
	N		14	46.6	–		
	T		24	–	–		
	W		–	–	6		
Webster	D	0.11	3	41.9	–	June 12–13, 2013	46°09′23″N, 69°04′52″W
	N		11	22.2	–		
	T		14	–	–		
	W		–	–	8		

The *F*_ST_ estimates are based on SNP (single-nucleotide polymorphism) results published previously (Renaut et al. 2011). The core microbiota is represented by percent of shared sequences for each form in each lake

*D* dwarf whitefish, *N* normal whitefish, *T* total number of whitefish per lake, *W* Number of water samples

### DNA extraction, amplification, and sequencing of intestinal bacteria

Adherent bacterial DNA from the intestinal segment was isolated by rinsing the interior of the intestines three times with 3 ml of sterile 0.9% saline [59] and extracted using a modification of the QIAmp© Fast DNA stool mini kit (QIAGEN). In order to ensure efficient lysis of Gram-positive bacteria, temperature and digestion time were increased during the incubation steps. Moreover, to maximize DNA extraction, the volume of supernatant and all of the products used with the supernatant (Proteinase K, Buffer AL, and ethanol 100%) were doubled. Thus, 1200 μl were transferred into the column (in two subsequent steps) and bacterial DNA was eluted from the column with 100 μl of ultrapure water (DEPC-treated Water Ambion®). Bacterial DNA from the water samples was also extracted using a modified QIAmp© Fast DNA stool mini kit (QIAGEN) protocol. The 0.22-μm membranes were transferred with a 1-ml InhibitEX buffer to bead beating tubes (Mobio), incubated overnight at 50 °C, and then vortexed for 1 h. The same modified protocol used for the adherent bacterial DNA was used. In order to test the sterility during the extraction manipulation, seven blank extractions were done with buffer only. Moreover, the same extraction kit was used between fish microbiota and water bacterial community in order to avoid bias during extraction. Extracted DNA was quantified with a Nanodrop (Thermo Scientific) and stored at − 20 °C until use.

The partial DNA fragments of bacterial 16S rRNA genes were amplified by touchdown PCR for adherent bacterial DNA. Touchdown PCR is the optimal method to avoid eukaryotic contamination, potentially due to cross amplification with host DNA [60, 61]. A region ~ 250 bp in the 16S rRNA gene, covering the V3–V4 regions, was selected to construct the community library using specific primers with Illumina barcoded adapters Bakt_341F-long and Bakt_805R-long [62] in a dual indexed PCR approach. The touchdown PCR of adherent bacterial DNA used 25 μl of NEBNext Q5 Hot Start Hifi PCR Master Mix, 1 μl (0.2 μM) of each specific primer, 15 μl of sterile nuclease-free water, and 8 μl of DNA (around 170 ng/μL). The PCR program consisted of an initial denaturation step at 98 °C for 30 s, followed by 20 cycles at 98 °C for 10 s, 67–62 °C (touchdown PCR annealing step) for 30 s, and 72 °C for 45 s. After the initial touchdown PCR cycles, an additional 15 cycles were run at 98 °C for 10 s (denaturation), 62 °C for 30 s (annealing) and 72 °C for 45 s (extension), and a final extension of 72 °C for 5 min.

The PCR amplification for water bacterial DNA comprised a 50-μl PCR amplification mix containing 25 μl of NEBNext Q5 Hot Start Hifi PCR Master Mix, 1 μl (0.2 μM) of each specific primer, 21 μl of sterile nuclease-free water, and 2 μl of water bacterial DNA (around 5 ng/μL). The PCR program consisted of an initial denaturation step at 98 °C for 30 s, followed by 30 cycles, with 1 cycle at 98 °C for 10 s (denaturation), 56 °C for 30 s (annealing) and 72 °C for 45 s (extension), and a final extension of 72 °C for 5 min. Negative and positive controls were included for all PCRs. All the PCR results, including the negative controls, were purified using the AMPure bead calibration method. The purified samples were quantified using a fluorometric kit (QuantIT PicoGreen; Invitrogen), pooled in equimolar amounts, and sequenced paired-end using Illumina MiSeq Bakt_341F-long and Bakt_805R-long at the Plateforme d’Analyses Génomiques (IBIS, Université Laval, Québec, Canada). To prevent focusing, template building, and phasing problems due to the sequencing of low-diversity libraries such as 16S rRNA amplicons, 50% PhiX genome was spiked in the pooled library.

### Amplicon analysis

Raw forward and reverse reads were quality trimmed, assembled into contigs for each sample, and classified using Mothur v.1.36.0 [63, 64]. Contigs were quality trimmed with the following criteria: (i) when aligning paired ends, a maximum of two mismatches were allowed; (ii) ambiguous bases were excluded; (iii) homopolymers of more than 8 bp were removed; (iv) sequences with lengths less than 400 bp and greater than 450 bp were removed; (v) sequences from chloroplasts, mitochondria, and non-bacterial were removed; and (vi) chimeric sequences were removed using the UCHIME algorithm [65]. Moreover, the database SILVA was used for the alignment and the database RDP (v9) was used to classify the sequences with a 0.03 cutoff level. The Good’s coverage index, Shannon index, inverse Simpson diversity, and weighted UniFrac tests were estimated with Mothur. The Good’s coverage index estimates the quality of the sequencing depth whereas alpha diversity (diversity within the samples) was estimated with the inverse Simpson index and the Shannon index. Beta diversity (diversity between samples) was calculated using a weighted UniFrac test [66], which was performed using thetayc distance.

### Statistical analyses

A matrix containing the number of bacterial sequences was constructed for each genus in each fish sample from the two Mothur taxonomy files (stability.an.shared and stability.an.cons.taxonomy). Therefore, OTUs (operational taxonomic units) with the same taxonomy were merged. This genus-merged matrix was used to perform the taxonomic composition analysis at the phylum and genus level, the principal coordinate analyses (PCoA), the permutational analysis of variance (PERMANOVA), the Metastats analysis, and the network analysis. Moreover, to determine if there was a significant difference at the alpha diversity level between species within and among lakes, we used a generalized linear model (GLM) with a Gaussian family followed by an ANOVA. In order to build the PCoAs, a Jaccard distance matrix was made from the genus-merged matrix after Hellinger transformation using the *vegan* package [67] in R (R Core Team 2016). The PERMANOVA analysis (number of permutations = 10,000) was also performed with the *vegan* package in R to test the species effects, the lake effects, and their interaction. The METASTATS software with standard parameters was also used (*p* ≤ 0.05 and number of permutations = 1000) to detect differential abundance of bacteria at the genus level between dwarf and normal whitefish [68]. Network analyses, based on a Spearman’s correlation matrix, were performed to document the interaction between dwarf and normal whitefish microbiota. The Spearman’s correlation matrix was calculated using R on the Hellinger transformed matrix. Moreover, *P* values and Bonferroni corrections were calculated for Spearman’s correlations for each sample. Then, the different networks were visualized using Cytoscape version 3.2.1, a software for visualizing networks [69]. Finally, PICRUSt (Phylogenetic Investigation of Communities by Reconstruction of Unobserved States, version 1.0.0) was used to predict putative functions for the whitefish microbiota based on the 16S rRNA sequence dataset [70]. To this end, our OTU data was assigned against the Greengenes database (released August 2013) and we used the Mothur command “make.biom” to obtain a data file compatible with PICRUSt.

## Results

### Sequencing quality

A total of 1,603,342 sequences were obtained after trimming for the entire dataset composed of 108 whitefish intestinal microbiota (44 dwarf and 64 normal whitefish) and 36 bacterial water samples (Additional file 1: Table S1). Among these sequences, 24,308 different operational taxonomic units (OTUs) were identified with a 97% identity threshold, representing 544 genera. The average Good’s coverage estimation, used to estimate the quality of the sequencing depth, was 99% ± 2% of coverage index.

Very few sequences were obtained from the five PCR negative controls (Additional file 2: Table S2). Although there were no bands after PCR amplification, 95 sequences in total were obtained from the five PCR negative controls, representing 0.006% of the total dataset. Sixty-one different species were identified with a range of 1–11 reads per bacterial species. Some of these sequences represented bacteria that are typically associated with fish, seawater, or freshwater environments, but also with fish pathogens (Additional file 2: Table S2). None were associated to humans or to the laboratory environment. This suggests that contamination was very low, but not completely absent, as typically observed in similar studies [71–73].

### Whitefish intestinal microbiota vs. water bacterial communities

**Fig. 1:**
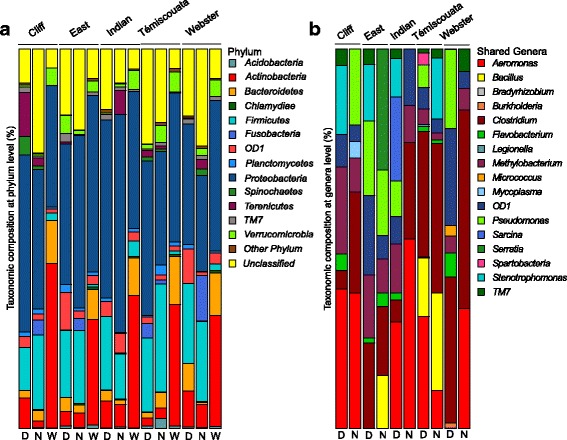
Taxonomic composition at the phylum and genus levels. **a** Relative abundance of representative phyla found in water bacterial communities and intestinal microbiota for dwarf and normal whitefish in each lake. This taxonomy is constructed with the database Silva and MOTHUR with a confidence threshold of 97%. **b** Relative abundance of genera observed in the core intestinal microbiota of dwarf and normal whitefish for each lake. In this study, the genera selected to constitute the bacterial core is present in 80% of the samples. *D* dwarf whitefish, *N* normal whitefish

Highly different communities at the genus level were observed with weighted UniFrac and PERMANOVA tests between the water bacterial community and whitefish microbiota within each lake and among the lakes (Table 2). Moreover, water bacterial communities as well as dwarf and normal whitefish microbiota had distinct dominant phyla composition (Fig. 1a). The water bacterial community was composed of *Proteobacteria* (38.7%), *Actinobacteria* (33.5%), *Bacteroidetes* (10.6%), *Verrucomicrobia* (4.4%), *OD1* (2.0), and *Firmicutes* (1.9%). The five most abundant phyla of dwarf intestinal microbiota were *Proteobacteria* (40.6%), *Firmicutes* (17.8%), *Actinobacteria* (6.1%), *OD1* (5.5%), and *Bacteroidetes* (3.4%), whereas the five most abundant phyla of normal microbiota were *Proteobacteria* (39.0%), *Firmicutes* (20.1%), *Fusobacteria* (4.1%), *Actinobacteria* (4.1%), and *Tenericutes* (2.5%). Thus, the phylum *Proteobacteria* dominated all sample types, but other phyla differed between the fish microbiota and water bacterial communities. Moreover, even if *Proteobacteria*, *Firmicutes*, and *Actinobacteria* were present in similar abundances between dwarf and normal microbiota, the phyla *OD1* and *Bacteroidetes* were more present in dwarf whitefish and the phyla *Fusobacteria* and *Firmicutes* were more present in the normal whitefish.

**Table 2:** Summary of weighted UniFrac and the PERMANOVA test statistics

Comparison	Lakes/effects	UniFrac	PERMANOVA		
		WSig	*F* value	R2	Pr (> *F*)
Both whitefish species microbiota-water bacterial communities (sequence dataset)	Water-whitefish	< 0.0010	33.834	0.185	< 0.0010
	Lakes	–	2.774	0.061	< 0.0010
	Water-whitefish*lake	–	1.278	0.028	0.074
	Cliff	< 0.0010	3.818	0.124	< 0.0010
	East	< 0.0010	6.910	0.210	< 0.0010
	Indian	< 0.0010	6.653	0.172	< 0.0010
	Témiscouata	< 0.0010	6.218	0.182	< 0.0010
	Webster	< 0.0010	7.341	0.269	< 0.0010
Dwarf-normal whitefish microbiota (sequence dataset)	Species	< 0.0010	2.273	0.019	0.002
	Lakes	–	2.812	0.096	< 0.0010
	Species*lake	–	1.493	0.051	0.0021
	Cliff	< 0.0010	1.931	0.081	0.006
	East	< 0.0010	1.821	0.092	0.019
	Indian	< 0.0010	0.913	0.037	0.530
	Témiscouata	< 0.0010	1.848	0.077	0.025
	Webster	< 0.0010	1.396	0.104	0.145
Dwarf-normal whitefish microbiota (PICRUSt results)	Species	–	0.448	0.003	0.697
	Lake	–	6.761	0.200	< 0.0010
	Species*lake	–	2. 273	0.067	0.016
	Cliff	–	0.152	0.007	0.958
	East	–	1.642	0.083	0.114
	Indian	–	0.413	0.017	0.793
	Témiscouata	–	5.052	0.186	0.019
	Webster	–	2.562	0.176	0.108

Three comparisons are shown: (i) comparison within and between lakes for the whitefish microbiota (dwarf and normal) and the water bacterial communities, (ii) comparison within and between lakes for the dwarf whitefish microbiota and the normal whitefish microbiota, and (iii) comparison of the PICRUSt results between dwarf and normal microbiota for all lakes combined and for each lake. For the three comparisons, we tested the lake effect and the interaction between the bacterial communities (water, dwarf, and normal whitefish) using PERMANOVA. UniFrac test is based on beta diversity and cannot be done with PICRUSt results

### Dwarf vs. normal whitefish microbiota: parallelism or not parallelism?

There was a significant difference between the dwarf and the normal whitefish microbiota at the genus level across all lake populations combined (Table 2). When treating each lake separately, the PERMANOVA tests revealed significant differences between dwarf and normal whitefish in Cliff, East, and Témiscouata lakes whereas no significant differences were found in Indian and Webster lakes (Table 2). Moreover, there is a gradient of genetic population distance between dwarf and normal whitefish from different lakes (Table 1) [56, 74]. Namely, sympatric whitefish from Cliff Lake are the most genetically differentiated (*F*_ST_ = 0.28) whereas those from Témiscouata Lake are the least differentiated (*F*_ST_ = 0.01). Thus, if there was some association between the extent of genetic divergence and the difference in microbiota, dwarf and normal whitefish from Cliff should have the most differentiated intestinal microbiota and Témiscouata should have the least differentiated ones. This was not the case as species specific microbiota was observed in the latter lake, whereas no significant difference was found in both Indian and Webster lakes where genetic differentiation between dwarf and normal whitefish is more pronounced (*F*_ST Indian_ = 0.06 and *F*_ST Webster_ = 0.11).

**Fig. 2:**
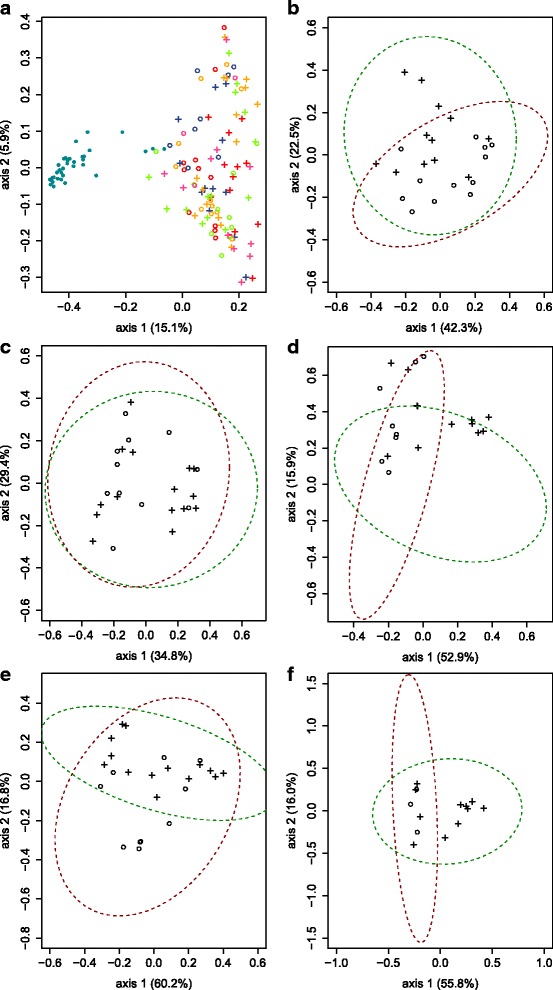
Principal coordinate analyses (PCoAs) of all the bacterial communities. These PCoAs are based on Jaccard index after a Hellinger transformation. **a** Comparison between water bacterial community and whitefish intestinal microbiota. Although the water bacterial communities come from five different lakes at different depths, all water samples are represented by a blue point. Each lake analyzed is represented by a different color: Cliff Lake (red), East Lake (blue), Indian Lake (orange), Témiscouata Lake (green), and Webster Lake (purple), and each whitefish species is represented by symbols: dwarf (circle) and normal (cross). **b–f** Comparison between dwarf and normal microbiota for each lake. Cliff Lake, East Lake, Indian Pond, Témiscouata Lake, and Webster Lakes are represented by **b**, **c**, **d**, **e**, and **f**, respectively. Each whitefish species is represented by different symbols: dwarf (circle) and normal (cross); ellipses of 95% confidence are illustrated and were done with dataEllips using R car package. The red and green ellipses represent the dwarf and normal species, respectively

The weighted UniFrac, which took into account the bacterial abundance rather than simply the presence or absence of taxa in the samples, were significant in all lake populations (Table 2). Therefore, the taxonomic composition of the microbiota was not always different between whitefish species depending on the lake but the abundance of microbiota always differed between whitefish species within each lake. No global differentiation was observed between whitefish species or lakes when all samples were included in the PCoA (Fig. 2a). However, the analysis revealed partially overlapping clusters corresponding to dwarf and normal whitefish in Cliff, East, Témiscouata, and Webster lakes (Fig. 2b, f). Dwarf and normal whitefish clusters were close to each other but nevertheless distinct. For example, in Cliff Lake, the dwarf cluster was more separated by axis one, whereas the normal cluster was more differentiated by axis two. In East, Témiscouata, and Webster Lakes, the opposite pattern was observed: dwarf and normal clusters were better separated by axis two and axis one, respectively. However, only three dwarf whitefish from Webster Lake could be collected resulting in low power of discrimination in that lake. Finally, dwarf and normal whitefish clusters almost completely overlapped in Indian Pond.

**Fig. 3:**
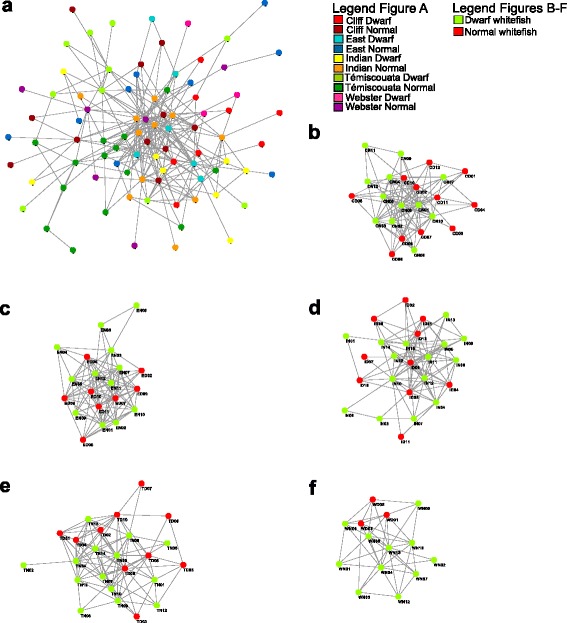
Network analysis of intestinal microbiota for dwarf and normal whitefish within- and between-lakes. The nodes represent a dwarf or a normal whitefish microbiota. The link (edge) between two samples highlights a Spearman correlation index and a significant *P* value corrected with Bonferroni correction. **a** Network analysis of whitefish microbiota among lakes. **b**–**f** Network analysis of dwarf and normal microbiota for each lake. Cliff Lake, East Lake, Indian Pond, Témiscouata Lake, and Webster Lakes are represented by the letter **b**, **c**, **d**, **e**, and **f**, respectively

Based on the network analysis, the five networks corresponding to each lake gave results that were similar to those obtained with the PCoA analysis, further supporting the observation that the dwarf-normal difference in microbiota varies according to the lake (Fig. 3). Although the network analysis containing all the fish samples revealed no clear pattern, lake-specific networks tended to cluster dwarf and normal samples separately in Cliff and Témiscouata Lakes. Even if the pattern is less clear for East Lake, the dwarf whitefish microbiota from this lake tended to cluster together (but not the normal whitefish microbiota). Also, no clear difference was observed in Indian Pond and as in previous analyses, interpreting patterns observed in Webster Lake was hampered by the small sample size of dwarfs, although microbiota of normal whitefish clustered together.

### Functional annotation of whitefish microbiota

**Fig. 4:**
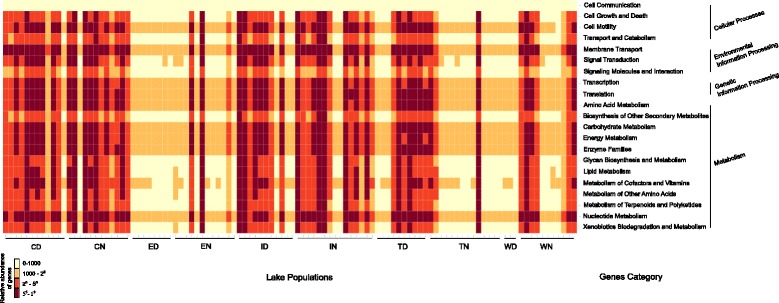
Heatmap of relative abundances of the most important metabolic pathways inferred by PICRUSt in the whitefish intestinal microbiota for each sample in all lakes. Gene category represented a set of genes with the same functional profile. Warm colors represent high abundances, and clear colors represent low abundances: *C* Cliff, *E* East, *I* Indian, *T* Témiscouata, *W* Webster, *N* normal whitefish, and *D* dwarf whitefish

Putative microbiota functions were predicted using PICRUSt by assignment of the predicted metagenome (Fig. 4). The gene category, which represented a set of genes influencing the same functional profile, varied widely according to the whitefish species or lake. Only one gene category, cell communication, was stable and had very low gene abundance. Some gene categories, including membrane transport, transcription, or energy metabolism, had high gene abundance in all dwarf and normal whitefish. However, the predicted microbiota functions revealed no significant functional differences between dwarf and normal whitefish microbiota within a given lake except for Témiscouata Lake (Table 2). Globally, there was no significant functional difference between dwarf and normal whitefish microbiota across all lakes combined. Instead, gene abundance differed among lakes and the interaction term between lake populations and species was significant, indicating a strong lake population effect but no significant functional differences between species (Table 2).

### Complementary analysis on whitefish microbiota: diversity, core intestinal microbiota, and Metastats

There was no difference between the dwarf and the normal whitefish in terms of bacterial diversity. Thus, the inverse Simpson index was not significant either between species within lakes or between lakes (Table 3). Similar results were also obtained using the Shannon index.

**Table 3:** Summary of GLM and ANOVA test statistics on the alpha diversity within- and between-lakes of whitefish species microbiota

Effect	*F* value	*t* value	*P* value
GLM + ANOVA			
Lake	0.833	–	0.507
Species	0.035	–	0.852
Lake*species	0.537	–	0.708
GLM			
Cliff	–	0.186	0.853
East	–	− 0.508	0.612
Indian	–	− 0.697	0.487
Témiscouata	–	0.478	0.633
Webster	–	− 1.240	0.218

These tests were performed with the inverse Simpson index, and similar results were observed with the Shannon index. Three effects are tested using a GLM followed by an ANOVA: the lake effect, the species effect, and their interaction

The core intestinal microbiota was defined as the microbial component shared by 80% of the samples. Three genera were shared among all the lake whitefish populations: *OD1*, *Methylobacterium*, and *Clostridium*. Additionally, all dwarf whitefish populations shared *Flavobacterium*, *TM7*, and *Pseudomonas*, whereas all normal whitefish populations shared *Aeromonas*. Within a given lake, more genera were shared between dwarf and normal whitefish, their number varying between four and 11 depending on the lake (Fig. 1b). Moreover, dwarf whitefish individuals shared more genera than normal whitefish did in Cliff, Indian, Témiscouata, and Webster Lakes. In East Lake, the same number of shared genera was observed between both species. Although the number of shared genera among populations of each species or among lakes was modest, they represented on average 49.5% of all dwarf whitefish shared sequences and 39% of all normal whitefish shared sequences (Table 1).

The Metastats analysis did not allow identifying any genera that were only present in one species. However, several genera were found in only one species within a given lake. These genera were blasted to identify the bacterial taxa being represented (Additional file 3: Table S3). Most of them were bacteria from the environment found in soil, plant, or freshwater. Interestingly, several bacteria previously found in seawater and human clinical specimens (but not found here in the negative control) were also found in intestinal whitefish microbiota, such as *Arsenicicoccus piscis*, *Lactococcus lactis*, or *Plesiomonas shigelloides* [75–77]. We also found bacteria known to be pathogenic in fish and humans, such as *Flavobacterium spartansii* and *Clostridium baratii* as well as *Bifidobacterium thermophilum*, which is a probiotic bacterium [78–80].

## Discussion

We investigated the intestinal microbiota of sympatric dwarf and normal whitefish pairs in order to (i) test for differences in whitefish intestinal microbiota and water bacterial community from the same lake, (ii) test for differences in intestinal microbiota between dwarf and normal whitefish from the same lake, and (iii) test for the occurrence of parallelism in those patterns. Below, we discuss the main results obtained for each of these objectives, as well as their relevance in the context of ecological speciation.

### Quality control

In order to improve the laboratory protocol and avoid bacterial contamination, meticulous care was taken by working in sterile conditions, performing blank extractions, using positive and negative PCR controls, and sequencing negative PCR controls. These controls revealed very few sequences in negative PCR controls (representing 0.006% of our dataset; Additional file 2: Table S2). These low-contamination sequences were typically associated with fish or fish environments and were represented, in a large majority, by one unique sequence. This contamination is therefore too low to influence the fish mucosa dataset and as such is unlikely to explain the lack of consistent parallelism observed in our dataset. Of the few previous studies that sequenced PCR negative controls, many found contamination without bands following PCR amplification [71–73]. Therefore, the PCR negative controls seemed not to be an adequate quality step and in order to know and reduce the risk of contamination, sequencing of PCR negative controls in the case of 16s rRNA gene pyrosequencing should be applied systematically, as we have done here.

### Whitefish microbiota vs. water bacterial community within a given lake

The whitefish intestinal microbiota was not reflective of the whitefish environment within each lake tested. Therefore, host physiology, immunity, and genetic background may play a role in determining the internal intestinal microbiota [34, 45, 47, 81]. The taxonomy between the fish intestinal microbiota and the bacterial water community was highly distinct among lakes. The water and the fish bacterial community shared 23, 21, 29, 27, and 23% of genera for Cliff, East, Indian, Témiscouata, and Webster lake populations, respectively. These values are substantially greater than the 5% shared OTUs reported recently between Trinidadian guppies (*Poecilia reticulata*) and their environment [45]. However, this could be due to the fact that these authors compared the fish microbiota with the bacterial community from both water and sediments. There are two major ways to colonize the fish intestine: via maternal microbial transmission [72, 82] or via the environment, which is the primary mechanism of microbiota acquisition for fish [83]. However, Smith et al. showed that the intestinal microbiota of three-spined stickleback (*Gasterosteus aculeatus*) tends to be more similar to food-associated bacteria rather than water-associated bacteria [48]. Although we did not sample the whitefish prey, our data demonstrate that around 25% of bacterial genera were shared between water and whitefish microbiota. Moreover, some of the main genera from whitefish microbiota were found at very low frequency in the environment. Therefore, even if the shared bacteria could come from the whitefish diet, it is quite likely that an important proportion of the intestinal microbiota could be attributed to the colonization of bacteria from the water.

### Whitefish intestinal vs. kidney microbiota and host effect

In this study, only the bacteria that formed a stable and specific association with the whitefish were analyzed. In fact, only the intestinal adherent microbiota of whitefish was selected, allowing for an indirect investigation of the host effect. In freshwater fishes, the dominant *Proteobacteria* is reported to be the most abundant phylum [38]. Also, the occurrence *Firmicutes*, *Bacteroidetes*, *Actinobacteria*, *Acidobacteria*, *Chlamydiae*, *Fusobacteria*, *Planctomycetes*, *Spirochaetes*, *TM7*, *Verrucomicrobia*, and *Tenericutes* has been reported in many freshwater fishes [38, 41, 42, 84, 85]. However, the phyla *OD1*, which was present at a relatively low frequency in both dwarf and normal whitefish, has usually been reported in freshwater samples but not freshwater fish, further supporting the acquisition of part of whitefish microbiota from the environment [86, 87].

Globally, we observed a total of 421 different genera in the intestinal mucosa from 108 fish. This is comparable to the level of diversity reported in other recent studies that analyzed 30 intestinal contents of five wild African cichlid fish species (tribe Perissodini) and 72 feces of the wild Amazonian fish tambaqui (*Colossoma macropomum*) that reported 121 and 525 genera, respectively [47, 88]. Therefore, the number of genera adherent to whitefish intestinal mucosa was similar to the number of genera found in feces or intestinal content in other wild freshwater fish. In a previous study of the kidney bacterial community in lake whitefish [49], the observed genera diversity (579 genera from 133 apparently healthy fish) was higher than that observed here for the intestinal mucosa. However, many more OTUs (24,308 OTUs) were found in the intestinal mucosa than in the kidneys (2168 OTUs). In both studies, mature fish were sampled in the same environment and they were sampled at the same period of time but in different years. The difference in genera diversity may result from both host genetic and immunity effects. Although the intestinal tract of animals contains the largest number of bacteria, which explains the difference between the intestinal mucosa and the kidney microbiomes at the OTU level, bacterial selection by the host may stabilize the number of intestinal genera [14, 16, 17, 81]. Such host-driven selection was highlighted in a zebrafish (*Danio rerio*) intestinal microbiota study, where the number of OTUs decreased during zebrafish development until reaching an equilibrium at fish maturity [89].

Interestingly, our data revealed no difference in diversity between intestinal microbiota of dwarf and normal whitefish found in sympatry within a given lake. This is in contrast with our previous study on kidney tissues where normal whitefish harbored a higher diversity than dwarf whitefish in all five lakes studied [49]. We had proposed that this difference may come from the distinct trophic niche of the two whitefish species. Dwarf whitefish feed almost exclusively on zooplankton [57, 58], whereas normal whitefish are generalists and feed on zoobenthos, molluscs, and fish prey [50, 58]. Moreover, Bolnick et al. observed a less diverse intestinal microbiota when the food was more diversified in both three-spined stickleback and Eurasian perch (*Gasterosteus aculeatus* and *Perca fluviatilis*), suggesting that the host had an effect on bacterial diversity [90]. Thus, the strikingly different diets between dwarf and normal whitefish had no apparent effect on the diversity of the adherent intestinal microbiota. As mentioned above, host genetic effects could select commensal bacteria in its intestine, which could perhaps explain the similar diversity level observed between dwarf and normal whitefish. Indeed, while the intestinal microbiota lives in a tight symbiotic relationship with the host, this is less so the case for kidney where the kidney microbiota has more of a pathogenic relationship with the host [16, 49]. Therefore, the comparison between symbiotic and pathogenic relationship could highlight the important host effect on the stabilization of the intestinal microbiota but not in the kidney.

Sequencing the microbial world has revealed an overwhelming intestinal microbiota impact on the host and has allowed documenting the core intestinal microbial communities in mammalian and teleost fish [3, 39, 40, 42, 45, 91–93]. The core intestinal microbiota corresponds to the OTUs or the genera shared among close host relatives and could be horizontally transmitted and/or selected as a common set of bacteria [3, 47]. For example, Roeselers et al. documented the occurrence of core intestinal microbiota between the domesticated and wild Zebrafish (*Danio rerio*) [42]. Here, our core microbiota data represented between 22 and 65% (mean ~ 44%) of genera shared between both species in each lake (Table 1). This percent of shared sequences is higher than that reported by Baldo et al., which found that the intestinal microbiota of cichlid species shared between 13 and 15% of sequences, but was equivalent to Sullam et al., which reported around 50% of shared sequences in the intestinal microbiota of Trinidadian guppy ecotypes [45, 47]. Therefore, the conservation of the core microbiota was strong within each whitefish species for each lake, further supporting the hypothesis of a strong host selective effect on its microbiota.

### No clear evidence for parallelism in intestinal microbiota between dwarf and normal whitefish

Parallelism is the evolution of similar traits in independent populations [94]. In the case of lake whitefish, the test for patterns of parallelism at many different levels may help identify the main factors that are at play in driving the process of ecological speciation in this system of repeated sympatric pairs. Here, given the many differences in their ecology and life history traits, we expected to observe some parallelism in differential intestinal microbiota between dwarf and normal whitefish species pairs. Indeed, parallelism between dwarf and normal whitefish has previously been documented for morphological, physiological, behavioral, and ecological traits [53, 55, 95–101]. Parallelism was also documented at the gene expression level, whereby dwarf whitefish consistently show significant overexpression of genes implicated with survival functions whereas normal whitefish show overexpression of genes associated with growth functions [56, 96]. Therefore, the apparent lack of parallelism in intestinal microbiota is somewhat surprising, especially given the known difference in trophic niches occupied by dwarf and normal whitefish. Indeed, fish diet is known to alter microbiota composition [83, 102–105]. Moreover, microbiotas have been reported to change in parallel with their host phylogeny [15, 17]. This phenomena coined “phylosymbiosis” has been reported in organisms as phylogenetically diverse as hydra, fish, and primates [40, 106, 107]. Here, we performed seven different types of analyses to test whether there were differences in the intestinal microbiota of the five whitefish species pairs that could have highlighted the occurrence of parallelism. However, while a clear difference between dwarf and normal whitefish microbiota composition was observed in three lakes, these differences were not parallel among lakes. Moreover, there was no difference between dwarf and normal whitefish from the other two lakes. Although the bacterial abundance (weighted UniFrac) differed between species in all five lakes, again, those differences were not parallel across lakes.

All in all, we found no clear evidence of parallelism in the intestinal microbiota across the five dwarf and normal whitefish species pairs. Instead, our results suggested that the main source of variation in whitefish microbiota was the lake of origin. As mentioned above, an important proportion of the intestinal microbiota could be attributed to the colonization by bacteria from the water. However, each lake studied had a distinct water bacterial community (PERMANOVA, water bacterial community of all the lakes = 0.0025). Although the whitefish host could modulate the intestinal microbiota, the lake bacterial variation could positively or negatively influence the intestinal microbiota of whitefish species. Cliff, Webster, and Indian lakes harbor the most genetically divergent species pairs, whereas East and Témiscouata species pairs are the least differentiated [51, 74]. These two groups of lakes are characterized by important environmental differences [108]. More specifically, lakes with the most divergent populations are characterized by the greatest oxygen depletion and lower zooplankton densities, suggesting harsher environmental conditions favoring more pronounced competition for resources between the two species [108]. On the contrary, lakes with the less divergent populations were characterized by more favorable environmental conditions [108]. Among the three lakes with the most genetically divergent species pairs, dwarf and normal whitefish differed in their intestinal microbiota only in Cliff Lake. East and Témiscouata species pairs (the two least differentiated populations) were also characterized by distinct intestinal microbiota. These observations suggest that while the lake of origin explains the composition of whitefish intestinal microbiota better than the species, there is no clear association between lake abiotic and biotic characteristics and the fish microbiota, suggesting that other factors that still need to be elucidated are at play.

### Whitefish microbiotas and their possible role in ecological speciation

Most of adherent bacteria living on the intestinal mucosa are not randomly acquired from the environment [90], but are rather retained by different factors in the host [16]. These symbiotic bacteria may play an essential role in the ecology and evolution of their hosts. Indeed, certain symbionts may affect evolutionary trajectories by conferring fitness advantages [26, 109]. For example, the microbiota of the desert woodrats (*Neotoma lepida*) enables its host to feed on creosote toxic compounds, suggesting a fitness advantage by limiting resource competition [26]. Symbionts can also influence speciation in several ways. First, there are two main processes that could influence pre-zygotic isolation: (i) microbe-specific, which involves bacterium-derived products such as metabolites and (ii) microbe-assisted, which involves bacterial modulation of the host-derived odorous products [14, 17]. In a recent study, Damodaram et al. showed that the attraction of male to female fruit flies is abolished when female flies are fed with antibiotics, implying a role of the fly’s microbiota in mate choice [22]. Second, symbionts can influence post-zygotic reproductive isolation with, for example, cytoplasmic incompatibilities leading to hybrid inviability [14]. These authors made crosses between two species of Nasonia wasp (*Nasonia vitripennis* and *Nasonia giraulti*) to create F2 hybrid larvae raised with their symbionts (conventional rearing) and without the symbionts (germ free). The F2 lethality was clearly more important with symbionts (conventional rearing) than without symbionts (germ free). Moreover, this lethality was not seen in pure larvae of both species reared with symbionts. Symbionts can also increase the host phenotype plasticity [109]. For example, a facultative endo-symbiotic bacterium called pea aphid U-type symbiont (PAUS) allowed the pea aphid (*Acyrthosiphon pisum*) to acquire a new phenotype: the digestive capability of alfalfa (*Medicago sativum*) [109]. This new phenotype supports a niche expansion that leads to geographic isolation between aphid populations and therefore indirectly confers a mechanism for pre-zygotic isolation. Given the absence of clear association between whitefish intestinal microbiota and whitefish species, it thus seems unlikely that any of these processes are at play in the speciation of the whitefish species pairs. This absence of parallelism across dwarf vs. normal whitefish microbiota highlights the complexity of the holobiont and suggests that the direction of selection could be different between the host and its microbiota.

## Conclusion

In summary, we analyzed the intestinal microbiota in the context of population divergence and speciation in the natural environments. We selected the whitefish mucosa; only the bacteria which formed a stable and specific association with the whitefish were analyzed. To our knowledge, this is the very first study which sequenced the intestinal adherent microbiota in natural fish host populations. Our main goal was to test for the occurrence of parallelism in the microbiota of dwarf and normal whitefish that evolved in parallel across different environments. However, no clear evidence for parallelism was observed at the bacterial level. We found distinct microbiota between the dwarf and the normal species in three of the five lake populations suggesting more selective pressure from the environment. This absence of parallelism across dwarf vs. normal whitefish microbiota highlighted the complexity of the holobiont and suggests that the direction of selection could be different between the host and its microbiota. Furthermore, the comparison of the adherent microbiota with the water bacterial environment and whitefish kidney bacterial community [49] provided evidence for selection of the adherent bacteria composition made by the host as well as bacterial diversity stabilization. Finally, an experiment without environmental variation would be useful to limit the effect of this in order to determine whether differences between whitefish species remain as large as observed here.

## Additional files

Additional file 1: **Table S1.** Steps used to reduce sequencing and PCR errors. (XLSX 9 kb)

Additional file 2: **Table S2.** Bacterial taxa found in the PCR negative control. (XLSX 19 kb)

Additional file 3: **Table S3.** Bacterial species specific to a single whitefish species within lake obtained with Metastats and BLAST. (XLSX 17 kb)

## Abbreviation

16 s rRNA: 16S Ribosomal RNA
ANOVA: Analysis of variance
CPA: Comités de protection des animaux
Fst: Fixation index
GLM: Generalized linear model
KO: KEGG Orthology
OTU: Operational taxonomic unit
PCoA: Principal Coordinates Analysis
PCR: Polymerase chain reaction
PERMANOVA: Permutational analysis of variance
PICRUSt: Phylogenetic Investigation of Communities by Reconstruction of Unobserved States
RDP: Ribosomal Database Project
YBP: Years before present
